# The Antisynthetase Syndrome: A Review

**DOI:** 10.3390/jcm15218308

**Published:** 2026-10-25

**Authors:** Evan Zyskowski, Shahrzad Abdollahi, Lawrence H. Brent

**Affiliations:** 1 Temple University Hospital, 3401 N. Broad St., Philadelphia, PA 19140, USA; 2 Jefferson-Einstein Medical Center, 5501 Old York Rd., Philadelphia, PA 19141, USA

**Keywords:** antisynthetase syndrome, myositis, interstitial lung disease, arthritis

## Abstract

Anti-synthetase syndrome (ASyS) is a relatively new and distinct clinical entity within the group of idiopathic inflammatory myopathies (IIM) characterized by the presence of autoantibodies targeting aminoacyl-tRNA synthetase (ARS), a family of enzymes responsible for protein translation but now recognized to possess important extracellular immunomodulatory functions. Anti-Jo-1 remains the most prevalent and studied antibody, but there exist additional antibodies, including PL-7, PL-12, EJ, OJ, KS, Zo, and Ha. These antibodies define a clinically similar but heterogenic syndrome with a variable combination of inflammatory myopathy, interstitial lung disease (ILD), inflammatory arthritis, and Raynaud’s phenomenon. Only a minority of patients initially present with multiple symptoms, whereas isolated pulmonary, muscle, or articular manifestations frequently predominate. Interstitial lung disease remains the predominant cause of morbidity and mortality, but neuromuscular involvement is central to disease pathogenesis and patient disability. Current evidence supports a concept of pathogenesis in which genetically susceptible individuals develop aberrant innate and adaptive immune responses from tissue injury, leading to persistent inflammation and progressive fibrosis. Despite advances in understanding ASyS, the disease remains associated with significant morbidity and mortality, largely driven by ILD. This review summarizes current evidence regarding pathogenesis, genetic susceptibility, clinical manifestations, diagnostic evaluation, and evolving treatment strategies for ASyS.

## 1. Pathogenesis of Anti-Synthetase Syndrome Materials and Methods

### 1.1. Initiation of Autoimmunity: Aminoacyl-tRNA Synthetases as Immunogenic Autoantigens

Current evidence supports a sequential model of pathogenesis, in which environmental triggers cause tissue injury and release of intracellular aminoacyl-tRNA synthetase (ARS) proteins into the extracellular space. In the lung, histidyl-tRNA synthetase (Jo-1) adopts a unique structural conformation, and cleavage by granzyme proteases generates highly immunogenic fragments capable of stimulating innate immune pathways. This may suggest the lung as the location of disease initiation. These proteins and fragments exhibit attributes, akin to chemokines, that recruit dendritic cells, monocytes, and NK cells and activate lymphocytes. Although the typical role of aminoacyl-tRNA synthetases is to link specific amino acids to their matching tRNA molecules during protein synthesis, several amino-acyl-tRNA synthetases have evolved cytokine- and chemokine-like activities capable of modulating immune responses. Jo-1 is the best known and most studied, although PL-7, PL-12, EJ, OJ, KS, Zo, and Ha define similar clinical syndromes, despite differing antigens. This suggests there may be a common pathogenic mechanism that exists across different antibodies even though their antigens differ [1,2].

### 1.2. Innate Immune Activation

#### 1.2.1. Chemokine-like Properties of Histidyl-tRNA Synthetase (Jo-1)

One of the most distinctive features of Jo-1 is its ability to function as a chemotactic agent independent of adaptive immunity. Howard and colleagues demonstrated that extracellular Jo-1 recruits activated CD4+ and CD8+ T lymphocytes, activated monocytes, and immature dendritic cells while interacting directly with chemokine receptors, particularly CCR5. Asparaginyl-tRNA synthetase (KS) similarly activates CCR3-mediated migration. This suggests that the release of ARS proteins from injured tissue amplifies local inflammation rather than the proteins serving as passive autoantigens [3].

#### 1.2.2. Pattern Recognition Receptors and Innate Signaling

Experimental murine models further demonstrate that soluble tRNA-synthetases alone induce inflammatory myositis without requiring exogenous adjuvants. Muscle inflammation appears to develop even in mice lacking functional T-cell receptors, B-cell receptors, or TLR4 signaling. Thus, aminoacyl-tRNA-synthetases can initiate inflammation through innate immune mechanisms independent of classical adaptive immune recognition. Additional experimental work demonstrates activation of both TLR2 and TLR4 pathways by immunogenic Jo-1 peptides, reinforcing the concept that innate immune sensing precedes adaptive autoimmunity [2,4,5,6].

### 1.3. Adaptive Immunity and Autoantibody Development

#### 1.3.1. CD4+ T Cells

Although innate immunity likely initiates the disease process, sustained tissue injury appears to require adaptive immune responses. Jo-1-specific CD4+ T cells have been identified in both peripheral blood and bronchoalveolar lavage fluid of patients with ASyS, demonstrating expansion of autoreactive helper T-cell populations at disease sites. These cells produce pro-inflammatory cytokines that support macrophage activation and B-cell differentiation, linking antigen recognition to chronic inflammation [6].

In addition to CD4+ T-cell activation, patients with ASyS exhibit significantly elevated circulating IL-17A concentrations compared with healthy controls, suggesting Th17 cell activation. IL-17 promotes the production of TNF-α, IL-1, IL-6, and IL-8 while facilitating communication between T and B lymphocytes. Importantly, IL-17 concentrations decrease following rituximab therapy, suggesting reciprocal interactions between B cells and Th17-mediated inflammation [7].

#### 1.3.2. B Cells and Autoantibody Production

Autoantibodies represent the defining immunologic feature of ASyS, yet increasing evidence suggests that B cells contribute beyond antibody production. Recent single-cell transcriptomic analysis demonstrates that circulating B cells exhibit a markedly activated phenotype characterized by interferon-responsive gene expression, oxidative stress pathways, altered cytoskeletal remodeling, enhanced antigen presentation, and increased expression of genes involved in cellular activation and migration. Memory B-cell subsets appear particularly dysregulated, suggesting that antigen presentation and maintenance of autoreactive immune responses are important functions beyond immunoglobulin secretion. These findings support the observed clinical benefit of B-cell depletion [8].

### 1.4. Cytokine Networks—Interferon Signaling

Multiple cytokine pathways appear to sustain chronic inflammation once adaptive immunity is established. Interferon signaling has emerged as a major immunologic signature in ASyS. Muscle biopsies demonstrate prominent HLA-DR expression together with transcriptional upregulation of interferon-γ-associated genes, supporting the activation of type II interferon pathways within affected skeletal muscle. Similarly, activated peripheral B cells demonstrate interferon-responsive transcriptional programs, indicating that interferon signaling operates systemically across multiple immune cell populations [8,9].

### 1.5. Natural Killer Cells and Lung-Centered Immunity

Several observations support the hypothesis that ASyS begins within the lung. Patients with active disease demonstrate reduced circulating NK-cell function accompanied by decreased expression of the activating receptor NKp30, despite preserved intracellular protein levels. Functional impairment correlates closely with diminished cytokine production and cytotoxic activity. Simultaneously, lung tissue demonstrates marked accumulation of NK cells, indicating redistribution from peripheral blood into inflamed lung tissue. This suggests NK-cell dysfunction contributing to defective immune regulation and persistent lung inflammation [2].

Additional support for a lung-centered origin comes from emerging microbiome studies demonstrating altered bronchoalveolar bacterial composition in anti-Jo-1-positive patients. Reduced abundance of *Veillonella* and altered correlations between microbial communities and inflammatory cell populations suggest that dysbiosis may influence pulmonary immune activation, although these findings remain preliminary [10].

### 1.6. Integrated Model of ASyS Pathogenesis

Current pathogenic models suggest genetically susceptible individuals encounter environmental or tissue-specific injury, particularly within the lung. Tissue damage exposes and proteolytically modifies aminoacyl-tRNA synthetases, which function as endogenous danger signals possessing intrinsic chemokine-like properties. These proteins recruit a host of immune cells while simultaneously activating innate immune pathways. Antigen presentation subsequently drives expansion of autoreactive CD4+ T cells and B cells, culminating in the production of anti-ARS antibodies. Sustained interferon signaling, IL-17-mediated inflammation, dysregulated NK-cell responses, and persistent B-cell activation perpetuate immune injury, resulting in chronic inflammation of skeletal muscle, skin, and lung with progressive fibrosis and tissue dysfunction [10,11,12].

## 2. Genetic Associations of Anti-Synthetase Syndrome

Anti-synthetase syndrome is recognized as a genetically heterogeneous autoimmune disease in which susceptibility, immune activation, and pulmonary phenotype are influenced by multiple genetic pathways rather than a single disease-causing variant. Current evidence supports contributions from immune regulatory genes, HLA alleles, and mucin-related genes involved in epithelial injury, fibrosis, and innate immune signaling pathways.

The strongest evidence for immune susceptibility implicates inflammatory cytokine signaling. Ponce-Gallegos and colleagues demonstrated that IL-1β polymorphisms are associated with an increased risk of ASyS, while another variant influences the circulating IL-1β concentrations, supporting enhanced inflammasome-mediated inflammation as a contributor to disease initiation [13].

Genetic predisposition also extends to adaptive immunity. In an anti-Jo-1 cohort, HLA-DRB1*03 and the linked HLA-DQA1*0501-DQB1*0201 haplotype were highly prevalent, consistent with prior observations that these alleles predispose to anti-Jo-1 formation. However, HLA status did not significantly affect organ involvement or overall clinical phenotype once disease manifestations developed, suggesting that HLA primarily influences susceptibility rather than disease expression [14].

Several studies have focused on genetic determinants of interstitial lung disease. Initial investigation of a MUC5B promoter variant found no association with overall ASyS susceptibility, ILD occurrence, anti-Jo-1 status, or usual interstitial pneumonia (UIP), despite its known role in idiopathic pulmonary fibrosis and rheumatoid arthritis-associated ILD. However, more recent work demonstrated that carriers of a GT genotype had a four-fold increase in the risk of developing UIP patterns. MUC5B may not be a universal susceptibility gene for ASyS, but it may identify a genetically at-risk subgroup for progressive fibrotic lung disease [15,16,17].

## 3. Clinical Features of Anti-Synthetase Syndrome

Although initially considered a subset of idiopathic inflammatory myopathies, contemporary evidence supports ASyS as a distinct clinical entity whose manifestations frequently extend well beyond skeletal muscle. Importantly, the classic triad of myositis, arthritis, and interstitial lung disease is present in only a minority of patients at disease onset, while isolated organ involvement is common and contributes substantially to diagnostic delay [18,19,20]. The essential clinical feature of ASyS is the presence of anti-synthetase antibodies. The prevalence of antisynthetase antibodies in patients with myositis and associated clinical features is shown in Table 1.

### 3.1. Evolving Clinical Presentation

The natural history of ASyS is characterized by the progressive accumulation of clinical manifestations rather than simultaneous disease onset. Large international cohorts demonstrate that only approximately 20% of anti-Jo-1-positive patients initially present with the complete clinical triad, whereas isolated myositis, arthritis, or ILD account for most initial presentations. Longitudinally, many patients subsequently develop additional manifestations, with ILD representing the most frequent new organ involvement. Similar findings have been reproduced across international cohorts and explain why many patients are initially diagnosed with polymyositis, dermatomyositis, rheumatoid arthritis, or idiopathic ILD before ASyS is recognized [18,20,25].

This evolving phenotype underscores the importance of maintaining clinical suspicion whenever patients present with otherwise unexplained myositis, inflammatory arthritis, or ILD, particularly when accompanied by Raynaud’s phenomenon, mechanic’s hands, fever, and positive anti-synthetase antibodies [19,26].

### 3.2. Pulmonary Manifestations

Interstitial lung disease (ILD) represents the hallmark extramuscular manifestation of ASyS and the principal determinant of morbidity and mortality. Across published cohorts, ILD develops in approximately 70–95% of patients during the disease course and can precede myositis by months or years. In many patients, pulmonary disease is the dominant or even isolated manifestation at presentation [27,28,29].

**Figure 1:**
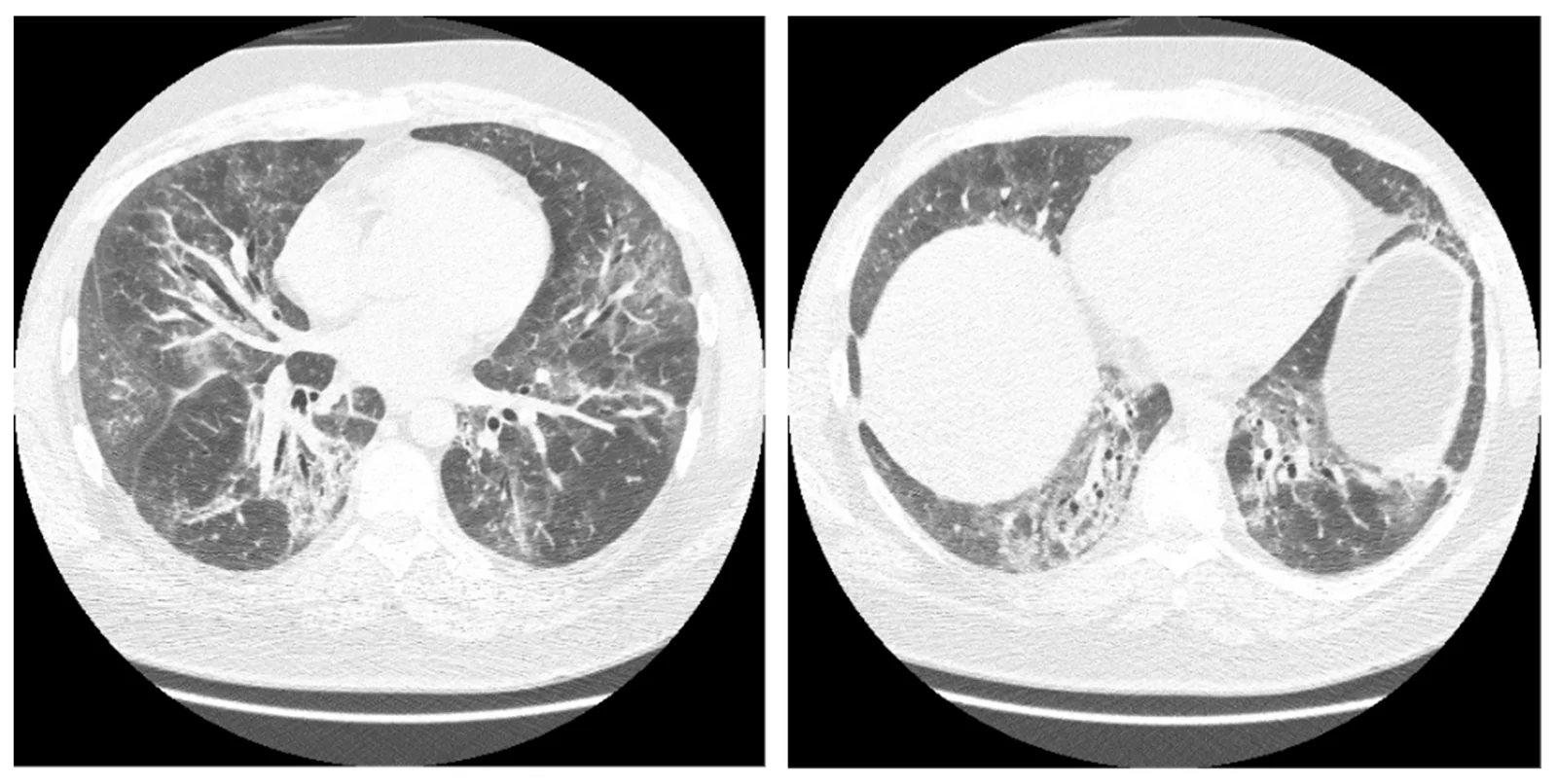
ILD with an NSIP pattern as seen in a patient with ASyS who is anti-Jo-1 positive with mechanic’s hands.

**Figure 2:**
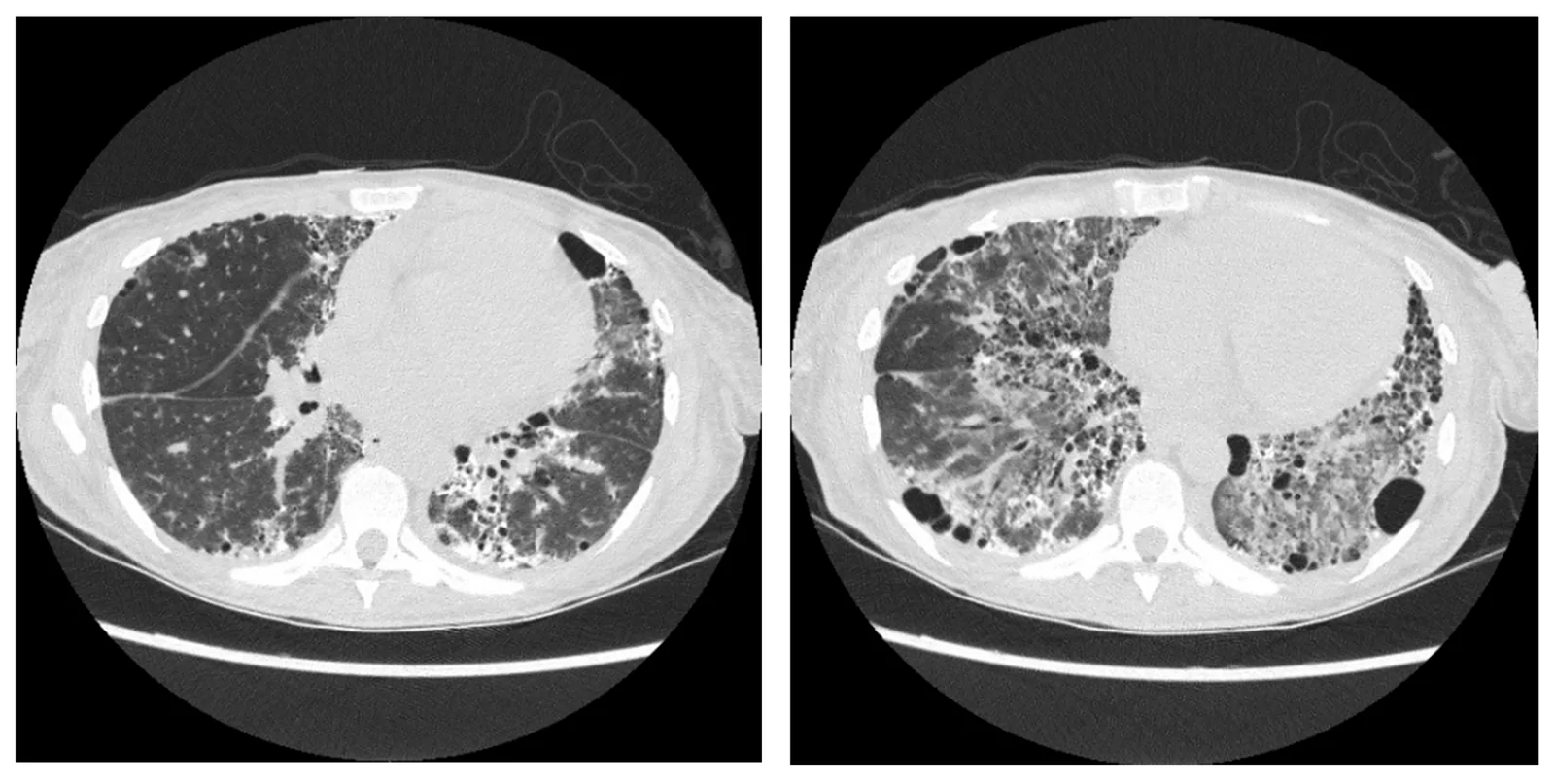
ILD with a fibrotic NSIP pattern in a patient with ASyS who is anti-Jo-1 positive with polymyositis.

**Figure 3:**
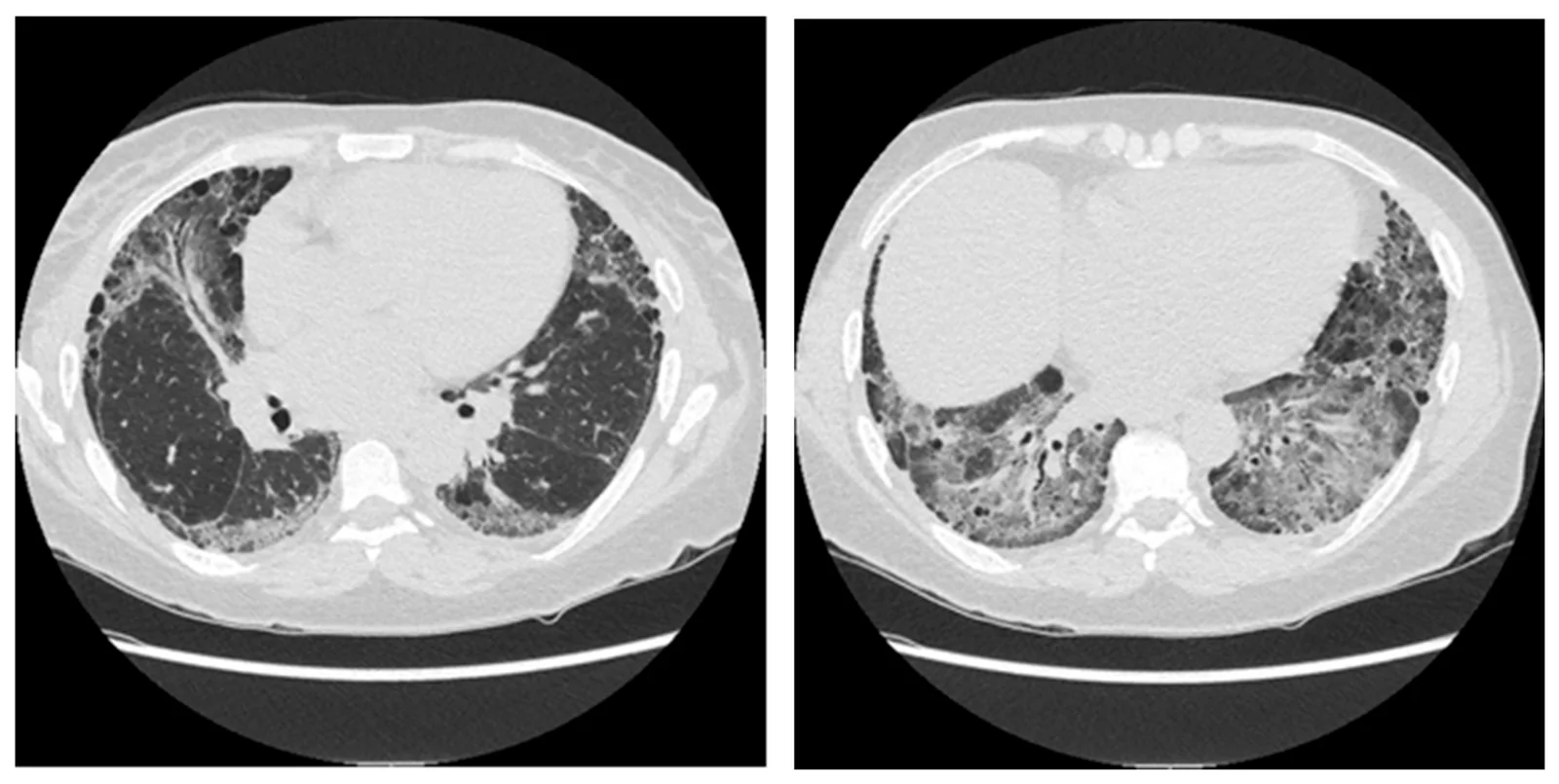
ILD with a fibrotic NSIP pattern in a patient with ASyS who is anti-PL-12 positive with polymyositis and Raynaud’s phenomenon.

The predominant radiographic pattern is nonspecific interstitial pneumonia (NSIP), and less frequently organizing pneumonia (OP). NSIP and OP may occur together on high-resolution computed tomography (HRCT). Usual interstitial pneumonia (UIP) occurs less commonly but is associated with poorer prognosis. Pulmonary function testing typically demonstrates restrictive physiology with disproportionately reduced diffusing capacity (DLCO), which correlates closely with radiographic disease extent [26,27,30]. Several patients with ASyS and ILD as seen on HRCT are shown in Figure 1, Figure 2 and Figure 3.

Clinical behavior is highly variable. Although Jo-1-positive patients, when compared with non-Jo-1-positive patients, have a better survival, mortality due to pulmonary disease (ILD and PAH) was similar in both groups [31]. Some patients experience slowly progressive fibrosis, whereas others develop rapidly progressive ILD (RP-ILD), characterized by respiratory deterioration within weeks to months. Recent multicenter studies have associated RP-ILD with male sex, fever, organizing pneumonia on HRCT, and pleural effusion, whereas severe muscle disease appeared less frequently in this phenotype. Fibrotic HRCT patterns, reduced DLCO, smoking history, and anti-Ro52 positivity have also been associated with poorer pulmonary outcomes [27,32,33]. Ro52 antibodies are found in patients with ASyS and are more frequent in patients with associated ILD, but some studies do not show them being associated with a worse pulmonary outcome [34]. It is not clear what the presence of Ro52 antibodies means for lung disease in patients with ASyS, though it can be associated with more severe involvement in other organ systems.

Pulmonary arterial hypertension (PAH) is a recognized pulmonary vascular complication of anti-synthetase syndrome and most commonly occurs in the setting of concomitant ILD. Along with ILD, PAH is a significant cause of mortality in ASyS patients [18,31]. In the University of Pittsburgh ASyS cohort with 202 patients, 14.8% had PAH, 11.5% in patients who were Jo-1 positive (*n* = 122), and 20.9% who were non-Jo-1 positive (*n* = 80) [31].

The ASPIRE Registry, a clinical registry of 1300 treatment-naive catheter-diagnosed PAH patients in the UK, tracked the survival data according to the major pulmonary hypertension groups and subgroups there from 2001 to 2010. The 3-year survival in Group 1 PAH was 63%, with scleroderma patients having the worst 3-year survival at 52%. Within Group 3, the ILD-associated patients had the worst 3-year survival at 16% [35]. These statistics should be heeded as their population preceded advances in diagnosis and treatments for PAH which are now available. Pulmonary vascular disease cannot be solely attributed to the severity of pulmonary parenchymal disease as the ASPIRE cohort demonstrated a small, but significant, subset of patients with pre-capillary pulmonary hypertension with minimal or no lung disease.

Echocardiographic abnormalities include elevated RV systolic pressure, RV and right atrial enlargement, and interventricular septal flattening. On right heart catheterization (RHC), pulmonary hypertension was associated with higher mean pulmonary artery pressure and pulmonary vascular resistance, lower cardiac output, and greater right-sided remodeling. Reduced right ventricular function was associated with lower DLCO, traction bronchiectasis, and use of supplemental oxygen. Pulmonary vascular pathology may therefore reflect both advanced ILD and intrinsic vasculopathy [22,31,36,37,38,39].

### 3.3. Skeletal Muscle Involvement

Although ASyS belongs to the inflammatory myopathy spectrum, muscle involvement is remarkably heterogeneous. Patients typically develop symmetric proximal weakness accompanied by elevated serum creatine kinase concentrations, myalgia, and variable dysphagia. Muscle biopsy frequently demonstrates perifascicular necrosis, now recognized as a characteristic pathological feature of ASyS distinct from classic polymyositis and dermatomyositis [11].

In the largest pathology-based cohort, all patients exhibited limb weakness, with approximately one-quarter demonstrating severe weakness, one-third exhibiting dysphagia or muscle atrophy, and one-third developing cervical muscle weakness. Fortunately, muscle disease generally responds well to corticosteroids combined with immunosuppressive therapy, and long-term neurologic outcomes are favorable compared with those of pulmonary disease [11].

Importantly, muscle involvement varies according to antibody specificity. Anti-Jo-1, anti-EJ, and anti-PL-7 antibodies are strongly associated with overt inflammatory myopathy, whereas anti-PL-12 and anti-KS frequently present with relatively mild or absent muscle disease despite severe pulmonary involvement. Anti-OJ and anti-Ha subsets appear capable of producing more severe myopathic phenotypes in selected patients [40,41,42,43].

### 3.4. Articular Manifestations

**Figure 4:**
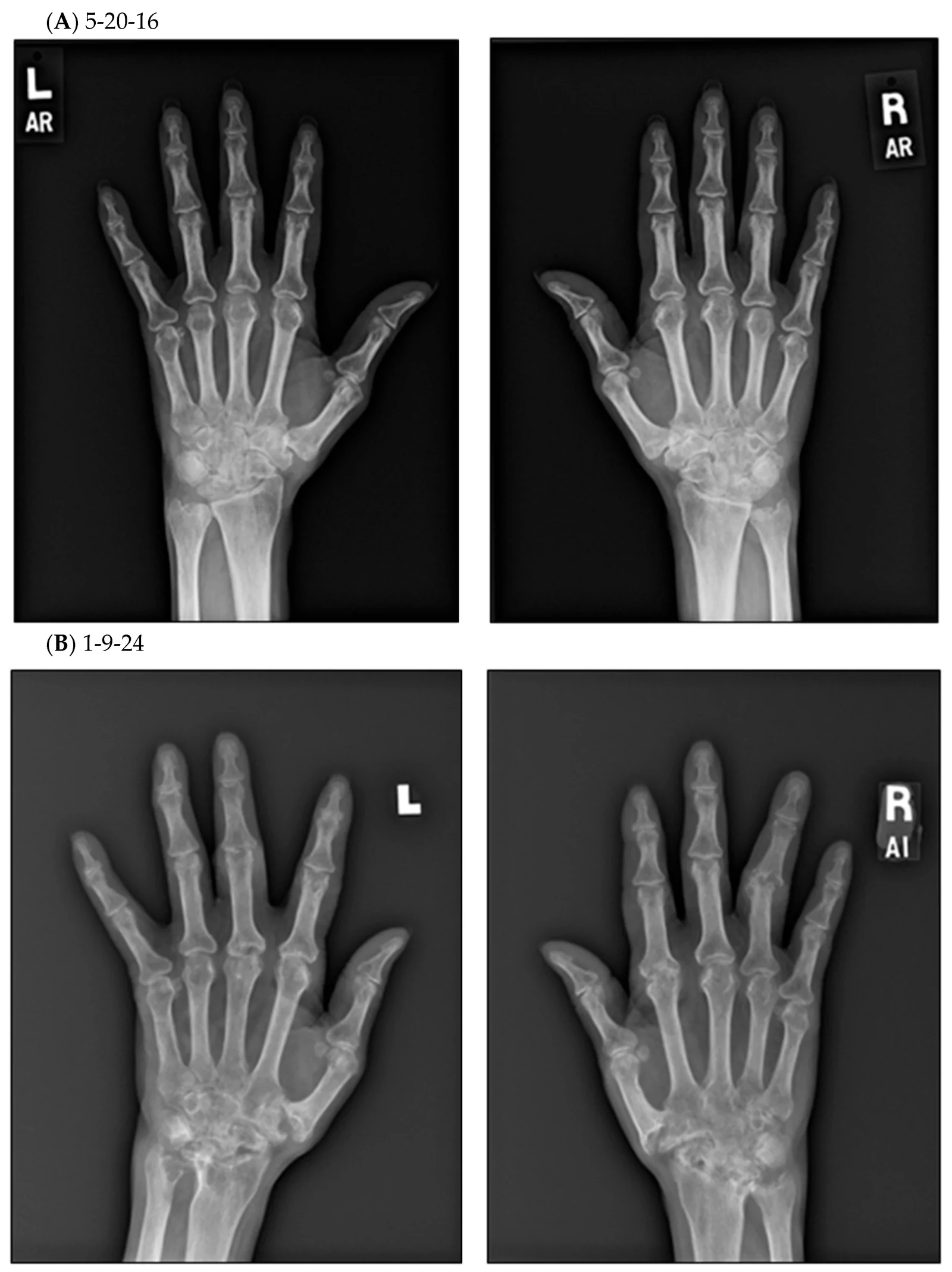
Progressive erosive arthritis in a patient positive for anti-PL-7 and negative for RF and CCP.

Inflammatory arthritis is one of the earliest and most common manifestations of ASyS, occurring in approximately 60–75% of patients. Arthritis frequently presents before muscle disease and often resembles rheumatoid arthritis with symmetric polyarthritis involving the small joints of the hands, wrists, and knees. Rheumatoid factor and anti-citrullinated protein antibodies may be present, and erosive disease occasionally develops, complicating differentiation from rheumatoid arthritis (RA). Some patients go on to carry diagnoses for both RA and ASyS. Erosive arthritic changes can be noted on radiographs, as seen in Figure 4 [40,43,44].

Arthritis phenotype does depend upon timing. Arthritis at disease onset more commonly resembles rheumatoid arthritis, whereas arthritis developing later during follow-up tends to display non-erosive joint pains accompanied by Raynaud’s phenomenon, fever, and mechanic’s hands. These observations suggest evolving immune mechanisms during disease progression [44].

### 3.5. Cutaneous and Vascular Manifestations

**Figure 5:**
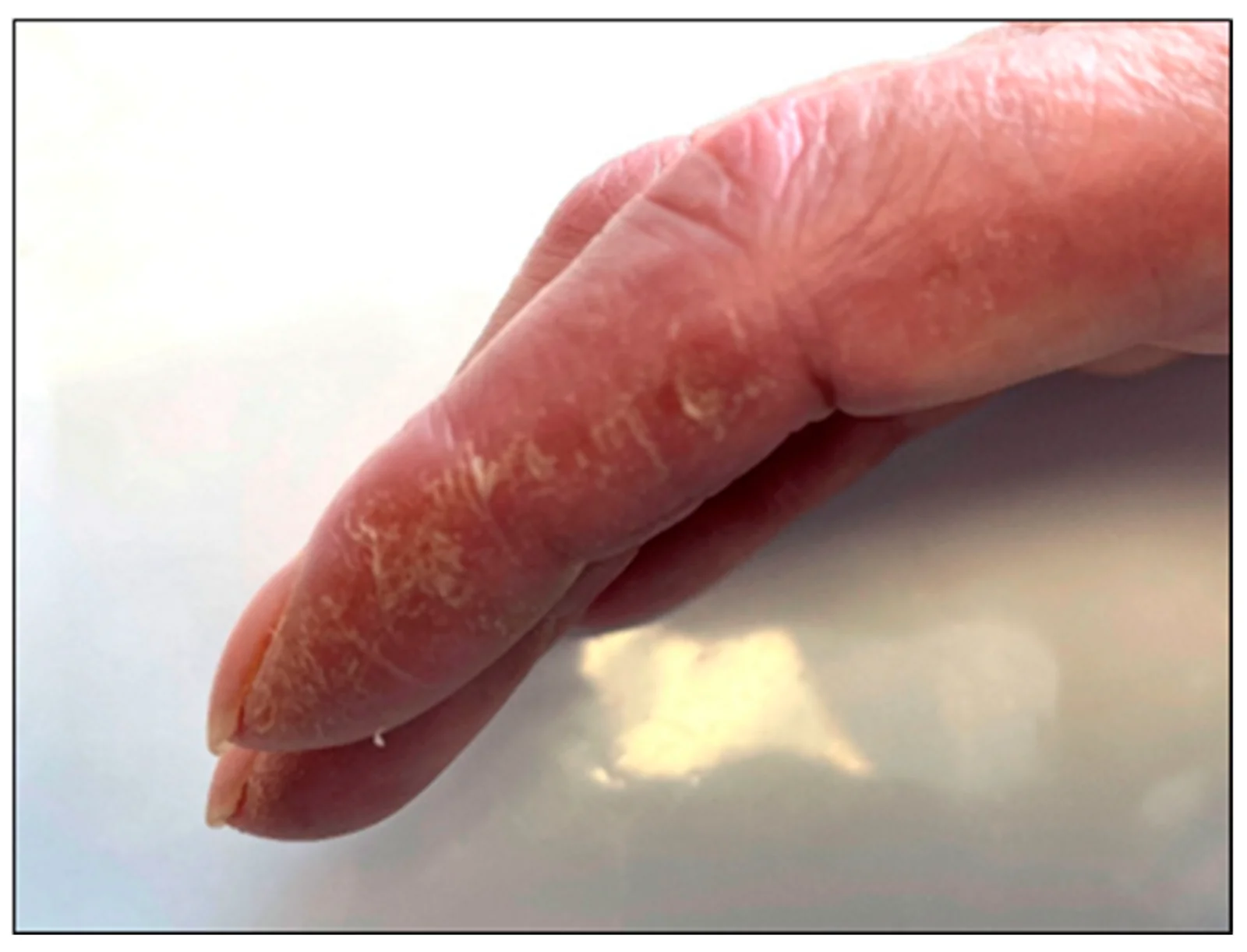
Mechanic’s hands of ASyS; typically involves the radial aspect of the second digit with erythema, scaling, and cracking of the skin. A similar rash may involve the feet (hiker’s feet). Image courtesy of Chester V. Oddis.

Mechanic’s hands (Figure 5) remain one of the most recognizable cutaneous manifestations of ASyS and consist of hyperkeratotic, fissured, scaling lesions involving the radial surfaces of the fingers and palms [45]. Although highly characteristic, mechanic’s hands are neither universally present nor entirely specific for ASyS. Recent work has expanded this spectrum to include “hiker’s feet,” a similar hyperkeratotic eruption involving the plantar aspect of the feet, frequently occurring simultaneously with mechanic’s hands and potentially improving diagnostic recognition [26,36].

**Figure 6:**
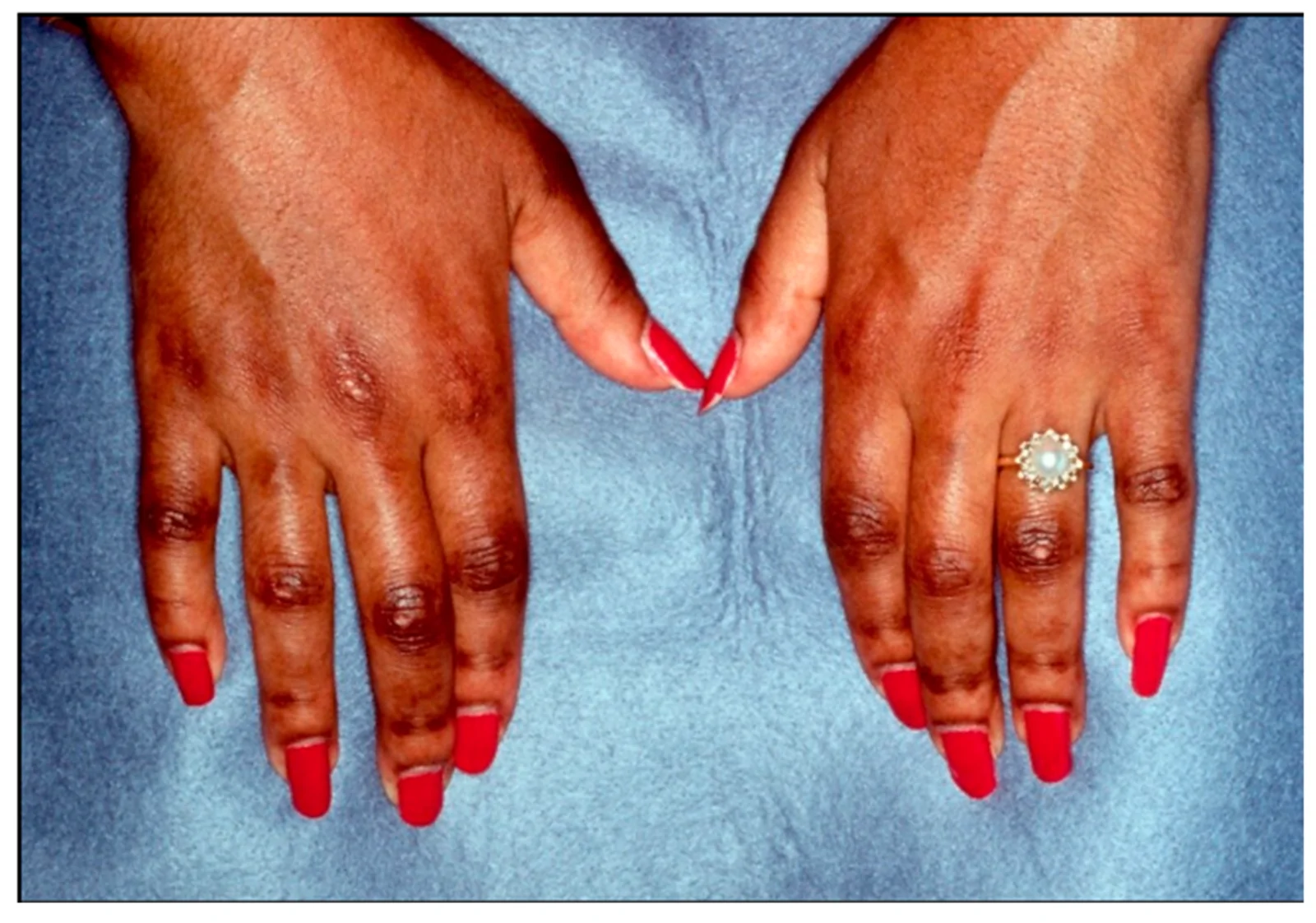
Gottron papules of dermatomyositis. An erythematous, scaly rash over the joints of the hands. This may also involve the elbows and knees.

Dermatomyositis-type cutaneous findings, including Gottron papules (Figure 6), heliotrope rash, V-sign, and shawl sign, occur in approximately one-third of ASyS patients, illustrating substantial overlap with dermatomyositis while preserving distinct systemic features such as ILD, arthritis, and mechanic’s hands. Data concerning the association of ASyS and malignancy are inconsistent, with some studies showing no association of ASyS with an increased risk of malignancy [46,47], while other data show an increased risk of malignancy [48,49], and may indicate a stronger association with anti-OJ antibodies [47].

Raynaud’s phenomenon occurs in approximately one-third to one-half of patients and frequently accompanies microvascular abnormalities detectable by nailfold capillaroscopy. Large multicenter studies demonstrate abnormal capillary architecture in over 60% of patients, including enlarged capillaries, hemorrhages, and reduced capillary density. Scleroderma-like capillaroscopic patterns are particularly associated with anti-Jo-1 positivity and longer disease duration. These changes may occur even in the absence of Raynaud’s phenomenon. Studies further demonstrate that nailfold capillaroscopy abnormalities vary according to anti-synthetase antibody subtypes, with anti-KS-positive patients exhibiting relatively mild microvascular disease [50,51].

### 3.6. Cardiac and Other Systemic Manifestations

Although it is less common than pulmonary disease, cardiac involvement is being increasingly recognized. There is evidence to suggest that myocarditis may be more prevalent than previously thought, with one small cohort study suggesting almost one-third of ASyS patients having evidence of myocarditis. The study had 43 patients, at a single center, 13 of whom showed evidence of cardiac involvement with cardiac magnetic resonance imaging abnormalities, elevated cardiac biomarkers, and elevated inflammatory markers. The study population was overwhelmingly female and over half of participants carried anti-Jo-1 antibodies. Modern cardiac magnetic resonance imaging incorporating T1/T2 mapping substantially improves diagnostic sensitivity when compared with conventional technologies. Pericardial involvement also occurs, particularly among anti-PL-7-positive patients. Gastrointestinal manifestations, such as dysphagia and esophageal dysfunction, reflect both skeletal muscle and smooth muscle involvement [26,52,53].

## 4. Influence of Anti-Synthetase Antibody Specificity

Although ASyS demonstrates a recognizable core phenotype, antibody specificity substantially influences disease expression. Anti-Jo-1 remains the most prevalent antibody and is generally associated prominently with myositis and arthritis. Anti-PL-7 and anti-PL-12 are more frequently associated with ILD-dominant disease and muscle involvement. Anti-EJ commonly presents with NSIP-predominant ILD that typically responds initially to immunosuppression but exhibits frequent relapse. Anti-OJ is associated with severe ILD, cardiovascular involvement, and a potentially increased risk of malignancy. Emerging anti-Zo and anti-Ha phenotypes likewise demonstrate the characteristic multisystem ASyS spectrum despite their rarity. Due to the scarcity of many of these antibodies, population-wide associations are difficult to ascertain [42,54,55,56,57].

## 5. Diagnostic Evaluation of Anti-Synthetase Syndrome

Because only a minority of patients present at disease onset with the complete clinical triad of ILD, myositis, and arthritis, diagnosis remains challenging and is often delayed. Evidence supports a multidisciplinary approach that integrates clinical assessment, serologic testing, pulmonary evaluation, imaging, and, when appropriate, histopathologic studies. Diagnosis of ASyS requires synthesis of these complementary modalities rather than reliance on any single test [57,58].

### 5.1. Clinical Recognition

Careful recognition of the diverse clinical features of ASyS is crucial to suspicion and initial diagnosis. ILD is frequently the presenting manifestation and may precede muscle disease by months or years, particularly in non-Jo-1 patients. Conversely, isolated myositis or inflammatory arthritis can dominate early disease, emphasizing that the absence of the classic triad should not exclude ASyS. Table 2 demonstrates the prevalence of ASyS manifestations as they relate to their antibody profiles. Recent analyses from the international CLASS project have further refined the clinical features most strongly associated with ASyS. Arthritis, muscle weakness, elevated muscle enzymes, ILD, mechanic’s hands, Raynaud’s phenomenon, fever, and pulmonary hypertension emerged as the principal clinical variables incorporated into classification criteria [58,59,60].

### 5.2. Serologic Evaluation

Serologic testing remains the cornerstone of diagnosis. Identification of an anti-ARS antibody helps establish the immunologic basis of disease and define the major antibody subtypes. The most notable tested antibodies include anti-Jo-1, PL-7, PL-12, EJ, OJ, KS, Zo, and Ha. Anti-Jo-1 remains the most common antibody, although non-Jo-1 antibodies frequently present with lung-dominated disease and greater diagnostic delay. Table 3 shows the antibody profiles of participants across multiple studies, reinforcing how much more common anti-jo-1 is to other antisynthetase antibodies. Quantitative antibody measurements may also provide complementary diagnostic information. Anti-Jo-1 titers correlate with creatine kinase levels, disease activity scores, and clinical evolution, suggesting a potential role in disease monitoring after diagnosis [42,64,65].

Commercial immunoblot and ELISA assays have substantially improved availability of ARS antibody testing; however, discrepancies remain when compared with RNA immunoprecipitation, the historical reference standard. Patients with ELISA-positive but RNA-immunoprecipitation-negative antibodies may still exhibit authentic ASyS phenotypes. Patients that had discrepant antibody testing between ELISA and immunoprecipitation were noted to have lower frequency of rash, arthritis, myositis, and mechanic’s hands. Discrepant results (positive ELISA, negative immunoprecipitation) were theorized to be due to ARS antibodies blocking the RNA binding site of ARS or reacting with denatured ARS. Thus, patients who may have a negative RNA immunoprecipitation result should be followed up with ELISA or protein immunoprecipitation testing [67,68].

Routine autoimmune screening should not rely solely on antinuclear antibody (ANA) testing. Conventional ANA demonstrates poor sensitivity in ASyS, and a negative ANA should never exclude the diagnosis. Instead, cytoplasmic staining on HEp-2 indirect immunofluorescence represents an important screening clue. Aggarwal and colleagues demonstrated that cytoplasmic staining possesses substantially greater sensitivity than ANA alone and that over 80% of ANA-negative ASyS patients demonstrate positive cytoplasmic staining. Combining cytoplasmic staining with anti-ARS antibody testing substantially improves diagnostic performance. More recent studies have shown that combining cytoplasmic staining patterns with low-titer nuclear ANA patterns further improves screening accuracy [69,70].

### 5.3. Pulmonary Evaluation

Interstitial lung disease is the principal determinant of morbidity and mortality. Baseline pulmonary assessment is mandatory and should include forced vital capacity (FVC), forced expiratory volume (FEV1), and diffusing capacity for carbon monoxide (DLCO). Six-minute walk testing is not mandatory but is routinely done in some clinics at baseline and upon follow-up visits. In ASyS patients, arthritis and myositis can negatively affect the outcome of the walking test. Serial measurements provide an objective assessment of disease progression and therapeutic response [71,72,73].

High-resolution computed tomography (HRCT) represents the imaging modality of greatest utility in the diagnosis and assessment of ILD. Characteristic findings include bilateral ground-glass opacities, reticulation, traction bronchiectasis, and consolidation with lower lobe-predominant disease. The most common radiographic patterns are nonspecific interstitial pneumonia (NSIP), organizing pneumonia (OP), and NSIP-OP overlap, whereas usual interstitial pneumonia (UIP) occurs less frequently but is more common among selected non-Jo-1 antibody subtypes. The most common ILD lung patterns, as seen on HRCT, can be seen in Figure 1, Figure 2 and Figure 3 [74,75,76].

Longitudinal imaging has become increasingly important for determining the trajectory of disease progress. Serial HRCT studies demonstrate that non-fibrotic inflammatory abnormalities frequently evolve toward fibrotic NSIP, whereas established fibrosis generally persists. Radiographic progression independently predicts reduced survival. Emerging quantitative imaging analysis (QIA) provides objective computer-assisted measurement of ILD burden and correlates with pulmonary physiology, patient-reported respiratory status, and overall disease activity, suggesting future utility as an imaging biomarker [77].

### 5.4. Evaluation of Myositis

Although myopathy may be clinically absent, assessment for occult muscle involvement remains essential. Serum creatine kinase, aldolase, lactate dehydrogenase, and aminotransferases should be measured, recognizing that muscle enzymes may remain normal despite active disease. Manual muscle testing (MMT-8), electromyography, and magnetic resonance imaging (MRI) complement the clinical examination when diagnostic uncertainty exists [71].

MRI using T2-weighted and STIR sequences identifies muscle edema, fascial inflammation, and fatty replacement while facilitating optimal muscle biopsy site selection. Recent investigations demonstrate that fascial high-signal intensity is particularly characteristic of ASyS and correlates with perifascicular muscle pathology [12,71].

### 5.5. Histopathology

Muscle biopsy is no longer routinely required in patients with classic clinical features and confirmatory serology but remains valuable in diagnostically challenging cases, particularly when antibody testing is negative or incomplete. On histopathologic evaluation, necrotizing myopathy is the predominant histologic pattern in anti-Jo-1-positive ASyS, frequently accompanied by macrophage inflammatory infiltrates and diffuse MHC-I expression [78].

More recently, HLA-DR immunostaining has emerged as a promising diagnostic biomarker. Perifascicular HLA-DR expression demonstrates high specificity for ASyS after exclusion of dermatomyositis and inclusion body myositis and reflects the activation of interferon-γ signaling pathways characteristic of the disease [9].

Lung biopsy is rarely necessary because HRCT combined with clinical and serologic findings usually establishes the diagnosis. When obtained, histopathology most commonly demonstrates NSIP, organizing pneumonia, or UIP depending upon disease stage and antibody subtype [74,79].

### 5.6. Diagnostic Criteria and Classification

Diagnosis of ASyS has been difficult to make since its inception as a separate clinical entity within the idiopathic inflammatory myositis taxonomy. Establishing the diagnosis generally consists of positive serum identification of a known associated antibody and the presence of classic manifestations. As seen in Table 4, early diagnostic criteria required the presence of at least one antibody and any combination of affiliated manifestations [80]. In comparison, Solomon placed increased weight on the presence of ILD and inflammatory myopathy as identifying features, whereas joint and skin involvement are considered minor criteria [81].

In 2024, a new research classification system was devised through expert opinion and data-driven consensus to identify key features and serologic markers of ASyS. The project investigated 2035 cases of ASyS and compared them to 2140 control cases from 92 centers across 30 countries worldwide. This recognized two levels of classification: probable and definite ASyS. Both require at least one of the core clinical features of the classic triad (arthritis, myositis, or lung disease). This system also assigned corresponding points to each identifier. Identification of an anti-synthetase antibody is required to be considered for “definite” ASyS. Set against a gold-standard dataset, sensitivity and specificity of definite ASyS (5.5 points) were determined as 94.3% and 99.7%, respectively, while the sensitivity and specificity measures for probable ASyS (5.0 points) sensitivity and specificity measures as97.5% and 87.5%, respectively. When compared against an independent validation dataset, definite ASyS had respective sensitivity and specificity values of 86.8%/99.2% and probable ASyS had 92.7%/87.3%. This new system can be seen in Table 5 [60,71,82].

## 6. Treatment of Anti-Synthetase Syndrome

Because randomized controlled trials remain scarce, current therapeutic approaches are derived largely from retrospective cohorts, case series, and expert consensus. Consequently, management is individualized according to disease severity, organ involvement, and treatment response [26,64,83].

High-dose glucocorticoids remain a cornerstone of therapy for many patients, particularly during flares. However, corticosteroid monotherapy is rarely sufficient to induce remission of symptoms or provide meaningful maintenance due to its long-term complications. Early introduction of steroid-sparing immunosuppressive agents is a common method by which to reduce cumulative glucocorticoid toxicity while improving long-term disease control. Conventional agents include azathioprine, methotrexate, mycophenolate mofetil, calcineurin inhibitors, and cyclophosphamide, with treatment selection guided by degree of pulmonary and muscle involvement [26,64].

Methotrexate and azathioprine remain commonly used first-line steroid-sparing agents predominantly for muscle disease. Comparative cohort analysis demonstrated similar improvements in muscle strength, creatine kinase normalization, corticosteroid reduction, and overall adverse event rates between the two therapies. Although methotrexate-associated pneumonitis remains a concern, it was rare and reversible following drug discontinuation [84].

Mycophenolate mofetil (MMF) is increasingly utilized as a steroid-sparing immunosuppressive agent in ASyS, particularly in patients with disease dominated by ILD. Although direct comparative prospective studies are lacking, retrospective studies generally show that MMF use is associated with stabilization or improvement of FVC, DLCO, dyspnea, and reduced corticosteroid use. Case reports show promise with MMF use as an additive or possible replacement for patients who have failed or had inadequate control of their disease with glucocorticoids or azathioprine [85,86,87,88]. More recent cases show further improvement in lung disease markers when combined with rituximab. Overall, the available evidence supports MMF as a reasonable steroid-sparing therapy, with a clear role in mild-to-moderate or chronic ILD [89,90,91,92].

Calcineurin inhibitors have demonstrated strong evidence for ILD stabilization. Long-term cyclosporine therapy in corticosteroid-refractory patients resulted in significant improvements in FVC, DLCO, and radiographic disease burden while allowing glucocorticoid reduction over prolonged follow-up [93]. Tacrolimus has also demonstrated similar efficacy, particularly when combined with corticosteroids. Multiple analyses have shown superior progression-free survival for initial corticosteroids with calcineurin inhibitor therapy compared with glucocorticoid monotherapy [94]. Tacrolimus may also be effective for muscle disease in patients with ASyS, but this has not been studied specifically in patients with ASyS and myositis [95].

Cyclophosphamide (CYC) remains an important induction therapy for severe or rapidly progressive ILD. Retrospective multicenter studies demonstrate improvement in pulmonary function after induction therapy, although its long-term efficacy appears inferior to rituximab in maintaining pulmonary progression-free survival beyond two years. Consequently, CYC is increasingly reserved for life-threatening disease or used as bridge therapy before maintenance immunosuppression [96].

Rituximab (RTX) has emerged as one of the best supported biologic therapies for refractory ASyS, particularly in patients with progressive ILD. In cases of rapidly progressive ILD (RP-ILD), rituximab is considered first-line therapy by the ACR/CHEST guidelines and is commonly used in combination with mycophenolate. Studies consistently demonstrate stabilization or improvement of pulmonary function, radiographic abnormalities, and muscle disease following B-cell depletion. Case series showed stabilization or improvement of severe ILD in two-thirds of treated patients, even after failure of CYC and other immunosuppressive agents. Measurable improvements in FVC, DLCO, HRCT abnormalities, and muscle strength are seen in long-term follow-up from six months to two years. This allowed corticosteroid dose reduction and discontinuation of supplemental oxygen in many cases. In comparison, CYC demonstrated equivalent short-term outcomes, but RTX may have superior two-year pulmonary progression-free survival. This distinction was made through observational association [96,97,98,99].

The RECITAL trial compared RTX to intravenous CYC in a mixed population of patients with connective tissue disease-associated ILD. At 24 weeks, the study was unable to show the superiority of RTX over IV CYC in terms of the primary endpoint of improvement in the FVC [100]. The limitations of the study were the heterogeneous population of CTD-ILD patients, the higher degree of severe ILD in its study population, and the use of monotherapy RTX in the study arm. In clinical practice, RTX is commonly used in conjunction with methotrexate or MMF.

Intravenous immunoglobulin (IVIG) has emerged as a useful adjunctive therapy, particularly in refractory myositis and ILD or patients unable to tolerate intensified immunosuppression. IVIG is FDA-approved for the treatment of dermatomyositis with efficacy in muscle and skin disease [101]. Most patients experience stabilization or improvement in pulmonary function, with significant reductions in corticosteroid requirements. Although infusion-related adverse effects were found to be relatively common, IVIG provides the advantage of immunomodulation without significant immunosuppression. Therapeutic plasma exchange has been reported as rescue therapy, in isolated cases of ILD refractory to corticosteroids and CYC, producing rapid reductions in anti-Jo-1 antibody titers and clinical improvement. Evidence remains limited to case reports for plasma exchange [62,66].

Targeted biologic therapies have become widely available, and their use has sparked interest in the treatment of ill-defined disease processes. Interleukin-6 blockade with tocilizumab produced dramatic and sustained remission across muscular, articular, and pulmonary manifestations in two patients with refractory disease who remained in remission for more than ten years [102]. It should be noted that the ACR conditionally recommends against the use of tocilizumab in progressive ILD in patients with idiopathic inflammatory myopathy-associated ILD. Similarly, interleukin-1 inhibition with anakinra demonstrated major clinical responses in highly inflammatory disease phenotypes, particularly those complicated by myocarditis [103]. Abatacept, targeting T-cell co-stimulation, has demonstrated encouraging improvements in DLCO, respiratory outcomes, and corticosteroid tapering in refractory ASyS-associated ILD [104].

Janus kinase (JAK) inhibitors are the latest evolving therapeutic approaches for refractory disease. Cohort studies demonstrate improvement in muscle strength, inflammatory markers, HRCT scores, respiratory symptoms, and glucocorticoid requirements in most refractory patients receiving tofacitinib or baricitinib. Case reports have shown successful treatment of rapidly progressive ILD requiring extracorporeal membrane oxygenation with tofacitinib in combination with tacrolimus and corticosteroids, as well as sustained remission of refractory ILD following baricitinib after failure of corticosteroids, IVIG, RTX, and CYC [105,106,107].

CD19-directed chimeric antigen receptor (CAR) T-cell therapy is emerging as an innovative therapeutic strategy currently under investigation for autoimmune diseases. Initial reports of patients with ASyS treated with CAR T-cell therapy demonstrate rapid normalization of muscle enzymes, restoration of muscle strength, improvement in pulmonary function, depletion of autoreactive B cells, and prolonged drug-free remission in patients with highly refractory disease after failure of multiple immunosuppressive agents, including rituximab. Subsequent reports have extended these observations to patients previously refractory even to plasma cell-directed therapies, suggesting that immune reprogramming may represent a viable future therapeutic option for refractory and severe ASyS [108,109,110,111].

Antifibrotic therapy is considered an adjunctive therapy to immunosuppression, rather than an alternative. Much of the evidence of its use stems from use in other diseases, such as idiopathic pulmonary fibrosis (IPF) and scleroderma-associated ILD, and centers around preservation of remaining lung function. Nintedanib has shown a decrease in FVC decline on its own and when combined with immunosuppression. Pirfenidone use has also been associated with improving FVC declines, but a paucity of real-world evidence of its effectiveness has limited its use. Both medications have proposed immunomodulatory effects, but there is a lack of meaningful in vivo evidence. Of note, their tolerability limits patient adherence, as gastrointestinal adverse effects affect up to two-thirds of patients. Their proposed use is in candidates with established ILD with radiographic or clinical progression who are on immunosuppression [112].

Overall, current evidence supports a stepwise treatment strategy beginning with glucocorticoids and early steroid-sparing immunosuppression, escalating to MMF, calcineurin inhibitors, RTX, and CYC according to pulmonary severity and disease progression. RTX currently possesses the strongest evidence among biologic therapies for refractory ILD, while IVIG serves as an important adjunct in selected patients. Emerging therapies, including JAK inhibitors, targeted biologics, and CAR T-cell therapy, offer promise for refractory disease but are supported primarily by observational studies and case reports. Table 6 provides an outline of the most used therapies and their intended use. The absence of adequately powered randomized controlled trials continues to limit evidence-based treatment recommendations [113].

## 7. Conclusions

Anti-synthetase syndrome is a heterogeneous autoimmune disease in which dysregulated innate and adaptive immune responses against aminoacyl-tRNA synthetases produce a multisystem disorder. Inflammatory myopathy remains a defining neuromuscular manifestation, but interstitial lung disease is the principal determinant of morbidity and mortality. Advances in understanding the immunopathogenesis of ASyS have refined the diagnostic strategies through improved serologic testing, advanced imaging, and identification of novel therapeutic targets. Current management emphasizes prompt use of glucocorticoids with early initiation of steroid-sparing immunosuppression. Escalation to biologic or targeted therapies is undertaken as needed for refractory disease. Nevertheless, treatment recommendations continue to rely on retrospective studies and expert consensus. Continued investigation into the molecular mechanisms underlying ASyS and the development of new therapeutic approaches will be essential to improving long-term outcomes for this complex syndrome.

## Figures and Tables

**Table 1:** Prevalence of antisynthetase antibodies in patients with myositis and associated clinical features [18,21,22,23,24].

Antibody	Prevalence in Myositis	Myositis	Arthritis	ILD
Anti-Jo-1	20–30%	82%	74%	82%
Anti-Pl-7	2–5%	80%	50%	77%
Anti-PL-12	2–5%	51%	42%	83%
Anti-EJ	2%	84%	53%	90%
Anti-OJ	1%	78%	44%	61%
Anti-KS	1%	-	-	-
Anti-Zo	<1%	-	-	-
Anti-Ha	<1%	-	-	-

**Table 2:** Clinical features associated with antibody profiles in patients with antisynthetase syndrome; number (%).

Antisythetase Antibody [24,61,62,63]					
Manifestation	All *n* = 1220	Jo-1 *n* = 767 (63%)	PL-7 *n* = 183 (15%)	PL-12 *n* = 131 (11%)	EJ/OJ *n* = 139 (11%)
Female	908 (74%)	568 (74%)	143 (78%)	96 (73%)	101 (73%)
Myositis	805 (66%)	569 (74%)	116 (63%)	53 (40%)	70 (50%)
ILD	998 (82%)	653 (85%)	151 (83%)	117 (89%)	118 (85%)
Arthritis	638 (62%) *n* = 1036	501 (73%) *n* = 691	69 (47%) *n* = 148	80 (76%) *n* = 105	33 (36%) *n* = 92
Raynaud’s	20 (10%) *n* = 208	16 (16%) *n* = 98	7 (18%) *n* = 40	4 (19%) *n* = 21	4 (11%) *n* = 36
Mechanic’s hands	167 (43%) *n* = 392	78 (45%) *n* = 174	38 (43%) *n* = 88	20 (43%) *n* = 47	31 (37%) *n* = 83
Ro52+	607 (53%) *n* = 1136	372 (51%) *n* = 731	86 (53%) *n* = 161	54 (44%) *n* = 122	74 (61%) *n* = 122

**Table 3:** Antisynthetase antibody profiles of patients with antisynthetase syndrome; number (%).

Antibody	Cavagna, 2019 [24] *n* = 828	Shi 2017 [61] *n* = 124	Zhao, 2022 [66] *n* = 111	Jiang, 2021 [62] *n* = 84	Total *n* = 1147
Anti-Jo-1	593 (72%)	62 (50%)	70 (63%)	36 (43%)	761 (66%)
Anti-Pl-7	95 (11%)	31 (25%)	17 (15%)	22 (26%)	165 (14%)
Anti-PL-12	84 (10%)	12 (10%)	9 (8%)	9 (11%)	114 (10%)
Anti-EJ	38 (5%)	19 (15%)	15 (14%)	12 (14%)	84 (7%)
Anti-OJ	18 (2%)	-	-	5 (6%)	23 (2%)
Anti-KS	-	-	-	-	-
Anti-Zo	-	-	-	-	-
Anti-Ha	-	-	-	-	-

**Table 4:** Early diagnostic criteria of anti-synthetase syndrome.

Connors et al., 2010 [80]	Solomon et al., 2011 [81]
Antisynthetase antibody plus one or more of the following:	Antisynthetase antibody plus two major criteria or one major and two minor criteria:
Myositis by Bohan and Peter ILD Arthritis Raynaud’s phenomenon Mechanic’s hands Persistent unexplained fever	Major Criteria Myositis by Bohan and Peter ILD Minor Criteria Arthritis Raynaud’s phenomenon Mechanic’s hands

**Table 5:** 2024 CLASS Project research classification criteria of Antisynthetase syndrome.

Patients Suspected of Antisynthetase Syndrome			
Classification Criteria for Antisynthetase Syndrome			
✓ Within each clinical/serological domain, only count the highest weight for calculating the score. Not present/not assessed/not available in any clinical/serological variable has score of 0.			
**Clinical Domain**	**Weight**	**Other Clinical Features**	**Weight**
**Joint Involvement**		**Mechanic’s Hands/Hiker’s Feet**	**1.5**
Inflammatory arthralgias or arthritis	**1.0**	**Raynaud’s Phenomenon**	**0.5**
**Muscle Involvement**		**Inflammatory Rashes** Gottron’s Heliotrope V-sign, Shawl sign Malar rash	**0.5**
Subclinical evidence of myositis (muscle enzymes/EMG/MRI) Clinical evidence of myositis Biopsy-proven myositis	**1.0** **1.5** **2.0**		
**Interstitial Lung Disease**		**Unexplained Fever**	**0.5**
UIP or other/unclassifiable pattern	**1.5**	**Serological Domain**	**Weight**
Predominant NSIP and/or OP	**2.5**	**Antisynthetase Antibodies**	
		Non-Jo-1+ by non-IP method Jo-1+ by any method, non-Jo-1+ by IP	**3.0** **3.5**
		ANA with cytoplasmic pattern	**1.5**
		Anti-Ro52 or anti-SSA antibodies	**1.0**

Definite ASyS = 5.5 points; probable ASyS = 5.0 points [82].

**Table 6:** Treatment algorithm for myositis-associated ILD. Adapted from Witt LJ, et al., 2016 [64] and Johnson SR, et al., 2024 [83].

First-line ILD therapy	Mycophenolate (preferred) Azathioprine Rituximab Calcineurin inhibitor (tacrolimus)
Additional options	Intravenous immunoglobulin JAK inhibitor Cyclophosphamide
Glucocorticoids	Short-term glucocorticoids

## Data Availability

No new data were created or analyzed in this study. Data sharing is not applicable to this article.

## Abbreviations

The following abbreviations are used in this manuscript:

ASyS	Antisynthetase syndrome
IIM	Idiopathic inflammatory myopathy
ILD	Interstitial lung disease
ARS	Aminoacyl-tRNA synthetases
NSIP	Nonspecific interstitial pneumonia
OP	Organizing pneumonia
UIP	Usual interstitial pneumonia
HRCT	High-resolution computed tomography
DLCO	Diffusing capacity
RP-ILD	Rapidly progressive ILD
PAH	Pulmonary hypertension
MMF	Mycophenolate mofetil
DMARD	Disease modifying anti-rheumatic drug
RTX	Rituximab
IVIG	Intravenous immunoglobulin
JAK	Janus kinase
CAR	Chimeric antigen receptor
